# A Comparative Analysis of COVID-19 Vaccines Based on over 580,000 Cases from the Vaccination Adverse Event Reporting System

**DOI:** 10.3390/vaccines10030408

**Published:** 2022-03-08

**Authors:** Kathleen Gallo, Andrean Goede, Cameron Mura, Renata Abel, Barbara Moahamed, Saskia Preissner, Susanne Nahles, Max Heiland, Philip E. Bourne, Robert Preissner, Michael Mallach

**Affiliations:** 1 Institute of Physiology and Science IT, Charité - Universitätsmedizin Berlin Corporate Member of Freie Universität Berlin, Berlin Institute of Health, Humboldt-Universität zu Berlin, 10117 Berlin, Germany; kathleen.gallo@charite.de (K.G.); andrean.goede@charite.de (A.G.); renata.abel@charite.de (R.A.); barbara.moahamed@charite.de (B.M.); michael.mallach@charite.de (M.M.); 2 School of Data Science and Department of Biomedical Engineering, University of Virginia, Charlottesville, VA 22904, USA; cmura@virginia.edu (C.M.); peb6a@virginia.edu (P.E.B.); 3 Department of Oral and Maxillofacial Surgery, Charité-Universitätsmedizin Berlin, Corporate Member of Freie Universität Berlin, Berlin Institute of Health, Humboldt-Universität zu Berlin, 10117 Berlin, Germany; saskia.preissner@charite.de (S.P.); susanne.nahles@charite.de (S.N.); max.heiland@charite.de (M.H.)

**Keywords:** COVID-19, vaccine safety, real-world evidence, carditis, thrombosis, Guillain–Barré syndrome

## Abstract

Background: The COVID-19 pandemic is being battled via the largest vaccination campaign in history, with more than eight billion doses administered thus far. Therefore, discussions about potentially adverse reactions, and broader safety concerns, are critical. The U.S. Vaccination Adverse Event Reporting System (VAERS) has recorded vaccination side effects for over 30 years. About 580,000 events have been filed for COVID-19 thus far, primarily for the Johnson & Johnson (New Jersey, USA), Pfizer/BioNTech (Mainz, Germany), and Moderna (Cambridge, USA) vaccines. Methods: Using available databases, we evaluated these three vaccines in terms of the occurrence of four generally-noticed adverse reactions—namely, cerebral venous sinus thrombosis, Guillain–Barré syndrome (a severe paralytic neuropathy), myocarditis, and pericarditis. Our statistical analysis also included a calculation of odds ratios (ORs) based on total vaccination numbers, accounting for incidence rates in the general population. Results: ORs for a number of adverse events and patient groups were (largely) increased, most notably for the occurrence of cerebral venous sinus thrombosis after vaccination with the Johnson & Johnson vaccine. The overall population OR of 10 increases to 12.5 when limited to women, and further yet (to 14.4) among women below age 50 yrs. In addition, elevated risks were found (i) for Guillain–Barré syndrome (OR of 11.6) and (ii) for myocarditis/pericarditis (ORs of 5.3/4.1, respectively) among young men (<25 yrs) vaccinated with the Pfizer/BioNTech vaccine. Conclusions: Any conclusions from such a retrospective, real-world data analysis must be drawn cautiously, and should be confirmed by prospective double-blinded clinical trials. In addition, we emphasize that the adverse events reported here are not specific side effects of COVID vaccines, and the significant, well-established benefits of COVID-19 vaccination outweigh the potential complications surveyed here.

## 1. Introduction

To immunize humans against COVID-19, resulting from an infection by severe acute respiratory syndrome coronavirus 2 (SARS-CoV-2), the U.S. Food and Drug Administration (FDA) has granted emergency use authorization to Moderna’s (mRNA-1273) and Janssen/Johnson & Johnson’s (Ad26.COV2.S) vaccines, while that of Pfizer/BioNTech (BNT162b2) has already received full approval. The Johnson & Johnson (J&J) vaccine relies on a single dose of a recombinant, replication-incompetent human adenovirus type 26 (Ad26) vector, encoding a full-length, membrane-bound spike (S) protein of SARS-CoV-2 [1]. Both the Pfizer/BioNTech and Moderna vaccines, which are each administered as two doses, rely on mRNA technology to encode the same spike protein, carried for delivery inside lipid nanoparticle capsules [2,3].

To date, a total of about eight billion vaccine doses have been administered, thus conferring acquired immunity against SARS-CoV-2 to a substantial number of individuals. An emerging concern is that, among those whom have been vaccinated, there have been several reported cases of moderate to severe side effects (such as might be expected for a vaccination campaign on this scale). In particular, vaccination has been associated with numerous cases of thrombotic events, such as cerebral venous sinus thrombosis (CVST; [4,5]). CVST, a severe and often fatal form of cerebral blood clot, forms in the junction of cerebral veins and larger, dural sinuses [6]. In the anatomical pathway by which blood exits the brain, via these sinuses, thromboses in the cerebral veins can prevent draining of the venous sinuses [6]; this increased pressure can lead to cerebral edema and, potentially, intracranial infarction and hemorrhage [6]. Among unvaccinated populations, CVST is known to afflict about 13 individuals per million per year [7].

Guillain–Barré syndrome (GBS), the most common and severe paralytic neuropathy, has also been associated with COVID-19 vaccinations [8]. Among unvaccinated populations, GBS affects about 100,000 people a year, and in approximately two-thirds of cases it is preceded by an infection roughly 1–3 weeks prior to the development of symptoms [9,10]. Infection by *Campylobacter jejuni*, *Mycoplasma pneumoniae* and several types of viruses (including cytomegalovirus, hepatitis E, Epstein–Barr and Zika virus) have been associated with the onset of GBS [10,11,12]. These pathogens are thought to trigger an immune response that results in the demyelination of, and subsequent damage to, peripheral nerves. Vaccines unrelated to COVID-19 have also been linked to triggering GBS [13]; for example, the risk of GBS after influenza vaccination has been found to be about one to three per million people [14,15].

In addition to CVST and GBS, some cases of myocarditis and pericarditis have been reported after COVID-19 immunization, specifically with the mRNA-based vaccines [16]. Myocarditis and pericarditis refer to the inflammation of the myocardium (i.e., heart muscle) and the pericardial sac (surrounding the heart), respectively; notably, these two ailments, as well as related conditions (e.g., myocardial infarction), often co-occur. Patients presenting with myocarditis/pericarditis after COVID-19 vaccination have had mild cases that generally resolved within a few days to few weeks. Post-vaccination myocarditis/pericarditis has been reported previously, most commonly after inoculation with the chickenpox vaccine [17].

A number of papers have examined the onset of adverse events, such as those described above, after COVID-19 vaccination. However, these recent studies have either (i) been limited to the reporting of incidence rates and otherwise lacking statistical analyses [18,19], (ii) unable to reach strong conclusions because of sparse data [20,21], or (iii) been unable to find statistically significant deviations in the occurrences of the adverse conditions described above [22,23]. To address these limitations, we have examined the number of cases of CVST, GBS, myocarditis or pericarditis after vaccination with either the Pfizer/BioNTech, Moderna, or J&J vaccines; in particular, we used the Vaccine Adverse Event Reporting System (VAERS), and the statistical properties of the occurrences of these post-vaccination side effects were quantified using odds ratios (ORs).

## 2. Materials and Methods

### 2.1. Data Extraction

Data for vaccination-associated side effects were downloaded from VAERS and converted to a MySQL database. For all reported vaccinations between 1 January and 24 September 2021, patient ages and genders were recorded, as was the identified manufacturer of the respective vaccine. Cerebral thrombosis cases were determined by doing a text search in the reported adverse reaction system, identifying all cases wherein a reaction contained the consecutive words “cerebral” and “thrombosis”; note that this search yielded hits with additional specifiers between the query terms, e.g., “cerebral venous thrombosis” or “cerebral venous sinus thrombosis”. Similarly, text searches were conducted using “myocarditis” and “pericarditis”. For GBS, the query “Miller-Fisher-syndrome” was included in the search terms, in addition to “Guillain-Barre syndrome”.

The results of the above searches were pruned by requiring that the adverse reaction occurred at most 14 days post-vaccination. Vaccination demographic data were obtained from the Centers for Disease Control and Prevention (CDC) website. The downloaded data contains total vaccination numbers for the U.S. until week 37 of our dataset timeline (i.e., 22 September), including vaccine manufacturer, age group, and gender of the respective patients.

In terms of background statistics, the prevalence of the various adverse events in the general population was assessed based on previously published studies concerning each condition (Table 1).

### 2.2. Statistical Analysis

To statistically characterize the risk of suffering a specific adverse event following a COVID-19 vaccination, ORs were calculated [24], using the statistical software R [25], which compare the risk of suffering an event for the ‘exposed’ (vaccinated) group relative to the ‘unexposed’ group (incidence rates in the general population). The incidence in the general population before 2020 serves as control group, consisting of people that reliably had not been exposed to a COVID-19 vaccination. Furthermore, the inclusion of VAERS reports until the end of September, when vaccines where readily available and not primarily distributed to the elderly population, ensures a close age and sex matching of both compared groups.

An OR exceeding one means that the exposure (i.e., vaccination) is statistically associated with higher odds of an adverse reaction, versus what would be expected by chance for the unexposed group. Numbers of counts for adverse event cases following a vaccination, and numbers for overall vaccination in the US population, were separated based on vaccine manufacturer, patient age, and gender. For each subgroup of interest, ORs were calculated using the average general prevalence of the respective adverse reaction.

ORs for the most relevant subgroups of the population (regarding age and gender) were calculated according to the number of vaccinations, which were administered for each of the three vaccines, and side effects that occurred for people of the chosen age and gender. Additionally, given prevalence studies of adequate numbers exist, 95% confidence intervals and *p*-values were calculated, where a *p*-value < 0.05 indicates a statistically significant difference from the expected value.

## 3. Results

### 3.1. Prevalence of Conditions in General Populations

To assess the general risk of suffering a CVST independent of COVID-19 vaccines, a number of studies and databases were examined, and the number of cases per million people/year was determined. The prevalence of reported cerebral thrombosis was found to range from two cases per million people/year up to 28.5 cases (Table 1A), with an average of 9.6 (using the largest number found, if the source included a range of values, e.g., the 2018 USA row in Table 1A). This background frequency was used as reference value for the calculation of ORs. Similarly, average incidence rates for GBS, myocarditis and pericarditis were assessed. For GBS, a global average of 13 incidences per million/year was determined; however, alternative values were also calculated, using the higher incidence rates from a Danish study (32; Table 1B). The reported incidence rates of myocarditis vary greatly with world region. Therefore, we took a global average of 200 per million/year; note that the bias towards the incidence rate in the US is in accord with the fact that the VAERS database includes mostly US numbers (Table 1C). For pericarditis, a mean of 130 per million/year was calculated, averaging over the available US, Finland, and Italy data (Table 1D).

### 3.2. Odds Ratios for Different Manufacturers, Gender and Age Groups

The absolute numbers of CVST cases for the three vaccine manufacturers that are approved in the US are comparable (Supplementary File S1). However, because the J&J vaccine has seen roughly 10-fold fewer administrations than other vaccines, the OR for cerebral thrombosis is vastly increased here. For the Moderna and Pfizer/BioNTech vaccines, the ratio nearly corresponds to the expected value of one, consistent with no vaccine-associated effects. The OR for the J&J vaccine is increased when considering only female patients, and even more so when the range is limited to women less than 50 yrs old. In contrast, the OR for CVST among men is lower (for both J&J and Pfizer/BioNTech), albeit still increased for the J&J vaccine relative to the overall, unvaccinated background population (Table 2A).

For Guillain-Barré syndrome, ORs for all three brands of vaccines are increased, and the effect is especially acute for the J&J vaccine (see the OR values > 10 in Table 2B). In addition, the ORs increase with greater patient age, and men are more frequently affected than are women (Table 2B).

For pericarditis and myocarditis, in general no increased risk could be assessed (Table 2C,D), apart from the category of men ages 18–24 yrs. For that group, (i) ORs are largely increased for the Pfizer/BioNTech vaccine, and (ii) Moderna also exhibits a statistically higher occurrence than expected, albeit to a lesser extent than for Pfizer/BioNTech (Table 2C,D).

**Figure 1:**
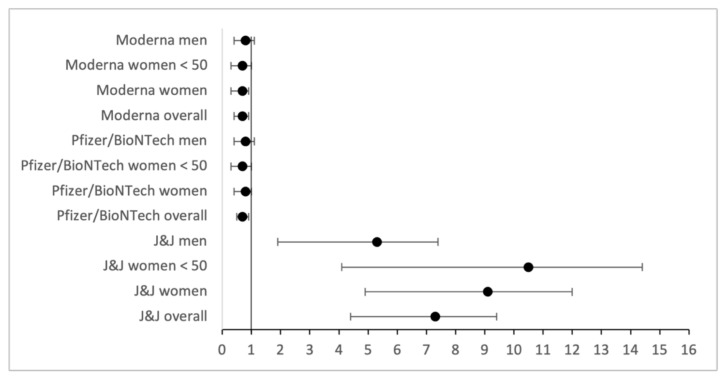
Odds ratios and confidence intervals for CVST, based on a general incidence of 13.2 [20].

**Figure 2:**
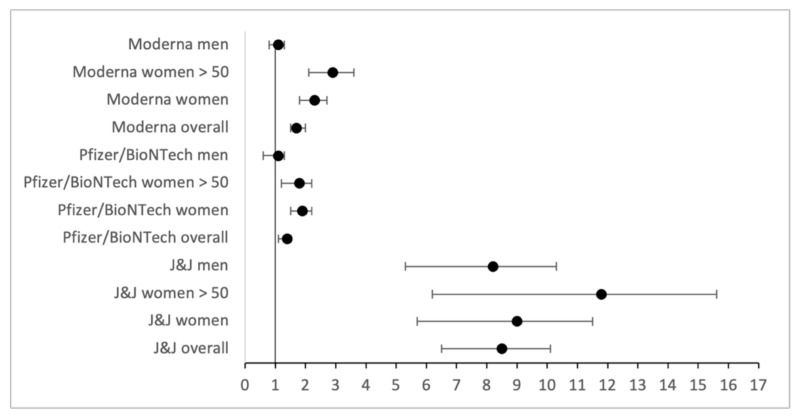
Odds ratios and confidence intervals for GBS, based on a general incidence of 17.7 [25].

In addition, 95% confidence intervals were calculated for adverse events with high ORs, using for both CVST and GBS the highest available incidence rate for which detailed numbers were available. For CVST, all confidence intervals for increased ORs are increased as well, and none of the ranges included an OR of one (Figure 1). This property also generally holds true for GBS too, with the exception of the data featuring only slightly increased ORs, specifically near OR ≈ 1.1 values for men (Figure 2).

**Figure 3:**
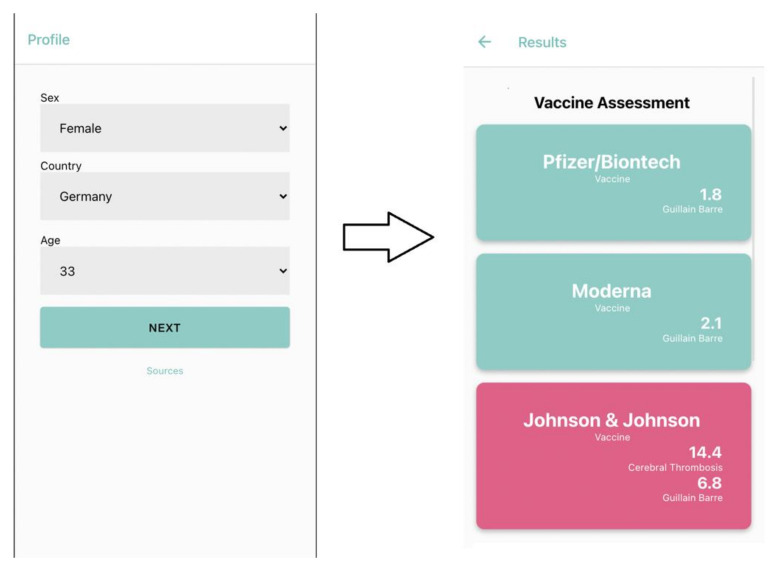
Integration of the statistical analysis into a freely available web application (screenshot shown), enabling the identification of a preferred vaccine based on one’s personal data.

In order to facilitate usage of the findings reported here, we developed a freely available web application (accessible via https://science-it.charite.de/vaccine/) that integrates the retrieved results and calculates personalized vaccine assessments based on user-provided age and gender (Figure 3). In addition, the application provides current infection data based on the supplied geographic location, and thus helps visualize needs for vaccination; this platform also simplifies the selection of an appropriate vaccine, given the availability of multiple choices.

## 4. Discussion

Overall, the cases we analyzed revealed an increased OR of CVST adverse events for the J&J COVID-19 vaccine, whereas for the Moderna and Pfizer/BioNTech vaccines, the frequency of cases was even lower than statistically expected based on the background/unvaccinated counts. This observation may well stem from the fact that not every case is reported to the VAERS database, while calculation of the expected frequency involves using the total number of vaccinations (i.e., no undercounting); therefore, the calculated ORs are lower-bound estimates, with the true numbers potentially being higher. On the other hand—especially as regards the higher thrombosis risk for (young) women—there could be other factors that play a role, such as hormone-based contraceptives or co-medications, which are not apparent or cleanly exposed in the analysis of VAERS data. Furthermore, even though the relative frequency of CVST cases is increased for the J&J vaccine, the absolute numbers are still low, with only 55 reported cases in over 14 million total vaccinations (Supplementary File S1).

Comparatively more adverse-event cases occurred for the Pfizer/BioNTech vaccines in connection with myocarditis, where almost 900 of the >1100 cases affected men, and 652 men younger than 25 years, leading to an OR greater than five for this vaccine (Table 2C). In contrast, only 20 total cases of myocarditis could be noted for the J&J vaccine, 13 of these being for male patients. The numbers for pericarditis reveal a similar picture, with the highest OR occurring for young men vaccinated with Pfizer/BioNTech.

The occurrence of GBS was statistically increased across all of the available vaccines and demographic groupings (gender, age), with higher frequencies being most pronounced for J&J, in general (regardless of grouping), and for administration of the Modern vaccine to older (>50 yrs) individuals.

It should be noted that (i) only a limited number of adverse events were analyzed, and (ii) the VAERS database is limited to vaccines approved in the U.S., meaning we were unable to examine any adverse side effects for the Oxford–AstraZeneca vaccine. Nevertheless, based solely on the results presented here, we can begin to draw some broad, overall conclusions. A general idea (and perhaps an emergent principle?) is that—given the possibility—the choice for a specific (booster) vaccine ideally would take into account a patient’s age and gender. So, for example, it might be advisable for younger women to use a COVID-19 vaccine for which (cerebral) thrombosis risks are comparatively lower, while younger men might have to take myocarditis risks into account when considering suitable vaccine options. Beyond patient gender and age, it may be generally beneficial to take as many other factors into consideration as possible if multiple different vaccines are potentially available; further analyses could focus on examining this possibility, including the potential statistical associations between (i) a unique individual (e.g., their genetics, metabolic profile, etc.), (ii) available vaccine options, and (iii) likelihoods of adverse reactions, including beyond those which were examined in this work. Finally, we reiterate that the adverse events examined here are well-established phenomena that occur both with infections (e.g., COVID-19) and, in some cases, even with other vaccines (see Introduction)—that is, these events are not specific side effects of various COVID vaccines. Any decision-making process, whether it concern population-wide policy or an individual/personal choice, will be healthiest if it is balanced and data-guided, weighing the potential complications surveyed here against the significant, well-established benefits of vaccination against COVID-19.

## 5. Conclusions

With the COVID-19 pandemic still ongoing and the continuous emergence of new variants, it will stay important in the future to vaccinate a large amount of the worldwide population. Therefore, assessments of safety and potential side effects of different vaccines continue to be an important topic.

In this paper, we laid focus on frequently reported side effects and evaluated a large number of public reports to perform a detailed analysis and assess the statistical significances. We could find statistically significant higher rates for each of the examined side effects, but only in specific subgroups of the population or depending on the administered type of vaccine. Therefore, we conclude that the choice for a specific vaccine should be made considering patient age and gender, given the availability of different vaccines, and further research has to be conducted to determine additional factors that might play a role in the occurrence of complications.

## Figures and Tables

**Table 1:** Evaluation of incidence rates for CVST, GBS, myocarditis, and pericarditis in the general population, either regionally or globally depending on the available data. References for the stated incidence rates are included in brackets.

Incidences per Million/Year (Citation/Source)	Publication Year	Region
A: Cerebral thrombosis		
2.4 [26]	2019	USA
14.5–28.5 [27]	2018	USA
13.2 [7]	2012	Netherlands
12.3 [28]	2008	Iran
2–5 [29]	2007	Great Britain
2.2 [30]	2001	Portugal
3.4 [31]	2001	Hong Kong
B: Guillain-Barré syndrome		
17.7 [32]	2019	Denmark
8.1–18.9 [33]	2014	Worldwide
13 [34]	2004	Worldwide
C: Myocarditis		
100–200 [35]	2021	Worldwide
102–1056 [36]	2017	Worldwide
104–400 [36]	2017	USA
D: Pericarditis		
78.3 [37]	2021	USA
33.2 [38]	2014	Finland
277 [39]	2007	Italy

**Table 2:** Overview of odds ratios.

	Johnson & Johnson	Pfizer/BioNTech	Moderna
A: Cerebral thrombosis			
Overall OR	10.0	0.9	1.0
OR Women	12.5	1.1	1.0
OR Women < 50	14.4	0.9	1.0
OR Men	7.3	0.6	1.1
B: Guillain-Barré syndrome			
Overall OR	11.6	2.0	2.2
OR Women	10.2	2.2	2.6
OR Men	13.0	1.7	1.8
OR Men > 50	17.1	1.9	2.5
OR Women > 50	13.4	2.1	3.3
C: Myocarditis			
Overall OR	0.2	0.7	0.4
OR Women	0.1	0.3	0.2
OR Men	0.2	1.1	0.7
OR Men < 25	0.1	5.3	1.9
D: Pericarditis			
Overall OR	0.6	0.7	0.5
OR Women	0.6	0.4	0.3
OR Men	0.7	1.0	0.7
OR Men < 25	1.3	4.1	1.7

## Data Availability

Link to VAERS: https://vaers.hhs.gov (accessed on 4 October 2021).

## Supplementary Materials

The following supporting information can be downloaded at: https://www.mdpi.com/article/10.3390/vaccines10030408/s1, Supplementary File S1: Cerebral thrombosis (CVST), Thrombosis, Guillain-Barre syndrome (GBS), Myocarditis, Pericarditis.
