# Supplementary material for: A Comparative Analysis of COVID-19 Vaccines Based on over 580,000 Cases from the Vaccination Adverse Event Reporting System
Source: Vaccines (Basel). 2022 Mar 8;10(3):408. doi: 10.3390/vaccines10030408 (PMC8950485; doi:10.3390/vaccines10030408)
Supplement: Supplementary file 1 [file vaccines-10-00408-s001.zip › vaccines-1594413-supplementary.pdf]

Supplementary Materials File S1:

Cerebral thrombosis (CVST):

Manufacturer	Overall OR	OR Women	OR Women < 50	OR Men
J & J	11.2	14.6	16.5	7.8
Pfizer/BioNTech	0.8	0.96	0.6	0.6
Moderna	0.8	0.7	0.94	0.85

Manufacturer	Overall Cases	Cases Women	Cases Women < 50	Cases Men
J & J	46	30	17	16
Pfizer/BioNTech	48	29	9	18
Moderna	37	17	11	20

Thrombosis:

Manufacturer	Overall OR	OR Women	OR Women < 50	OR Men
J & J	1.6	2	1.9	1.3
Pfizer/BioNTech	0.2	0.3	0.2	0.2
Moderna	0.2	0.3	0.2	0.2

Manufacturer	Overall Cases	Cases Women	Cases Women < 50	Cases Men
J & J	830	510	238	316
Pfizer/BioNTech	1466	872	355	583
Moderna	1103	646	236	457

Guillain-Barre syndrome (GBS):

Manufacturer	Overall OR	OR Women	OR Men	OR Men > 50	OR Women > 50
J&J	8.3	6.8	9.7	13.6	9.3
Pfizer/BioNTech	1.6	1.8	1.3	1.5	1.6
Moderna	1.8	2.1	1.4	2	2.5

Manufacturer	Overall cases	Cases Women	Cases Men	Cases Men > 50	Cases Women > 50
J & J	49	19	30	21	13
Pfizer/BioNTech	134	80	54	32	37
Moderna	117	71	44	33	43

Myocarditis:

Manufacturer	Overall OR	OR Women	OR Men	OR Men < 25
J & J	0.1	0.05	0.2	0
Pfizer/BioNTech	0.4	0.1	0.6	3.5
Moderna	0.25	0.08	0.4	1.3

Manufacturer	Overall Cases	Cases Women	Cases Men	Cases Men < 25
J & J	9	2	7	0
Pfizer/BioNTech	480	96	382	264
Moderna	247	40	206	93

Pericarditis:

Manufacturer	Overall OR	OR Women	OR Men	OR Men < 25
J & J	0.2	0.2	0.2	0.4
Pfizer/BioNTech	0.2	0.1	0.3	1.2
Moderna	0.15	0.08	0.2	0.5

Manufacturer	Overall cases	Cases Women	Cases Men	Cases Men < 25
J & J	26	12	14	3
Pfizer/BioNTech	352	91	258	161
Moderna	202	56	146	62

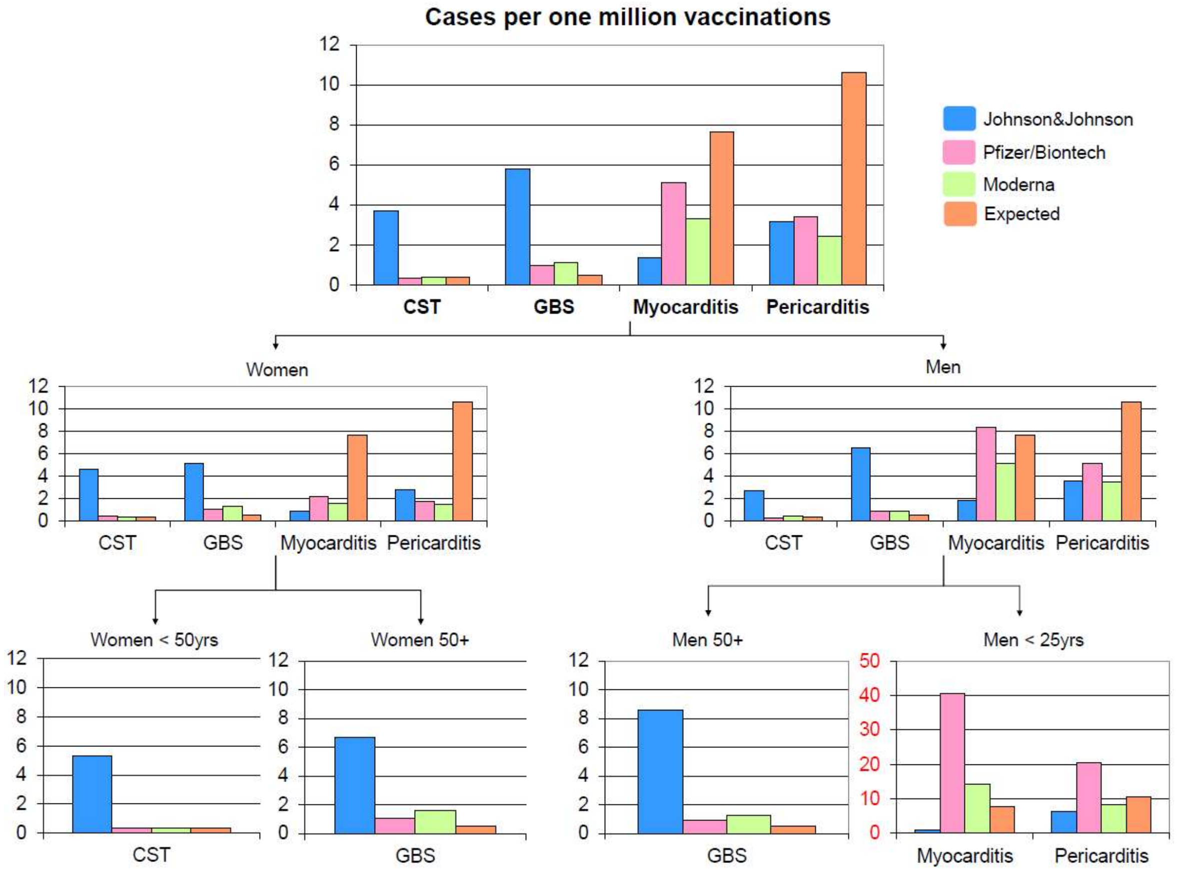

Supplemental Figure S1: Cases of cerebral venous sinus thrombosis (CST), Guillain-Barré-Syndrome (GBS), myocarditis and pericarditis per one million vaccinations: overview, women vs. men and age-dependent differences.
